# The Technique of the Tuberculin Test1Read before the Western Iowa Veterinary Medical Association, November, 1895.

**Published:** 1896-02

**Authors:** G. A. Johnson


THE JOURNAL
OF
COMPARATIVE MEDICINE AND
VETERINARY ARCHIVES.

VOL. XVII. *FEBRUARY*, 1896. NO. 2.

### THE TECHNIQUE OF THE TUBERCULIN TEST.[1]

BY G. A. JOHNSON, D.V.M.

THE subject of tuberculosis and its diagnosis is a subject that is demanding more and more attention from sanitarians the world over; and, as all veterinarians should be sanitarians, therefore they are more or less interested in the workings of the test.

It is the unanimous opinion of all leading veterinarians, based upon experiments which have fully demonstrated the fact, that the tuberculin test is the only practical and reliable method of diagnosing tuberculosis in the live animal.

But some may ask, What is the tuberculin test? In reply I would say that it is the physiological action of tuberculin when injected into the system in the proper quantities.

It is needless for me to take up your time with a discussion of the source, composition, and methods used in preparing tuberculin, as these are settled questions familiar to all of the profession.

The test is a thoroughly scientific procedure, very delicate and precise in its action, and, to be attended with success, must be conducted on purely scientific grounds. When properly conducted it is nearly if not quite infallible and of untold value, but when carelessly conducted it is not only misleading in its results, but may be the cause of the spread of the disease and loss of life, by diseased animals being passed as healthy, and of loss by healthy animals being condemned and destroyed, either of which places the test in a false light.

[1] Read before the Western Iowa Veterinary Medical Association, November, 1895.

Two essential factors that enter into the workings of the test are, first, the tuberculin, and secondly, the manner in which it is used. The tuberculin must be pure and of a reliable strength, or the results will of necessity be unreliable. Likewise, if it be not properly used the results will be misleading and uncertain.

Having secured a reliable preparation, the first step on arriving at the place is to determine what animals are to be tested. These should have their usual allowance of grain and water in the morning, and then be placed in the stable and marked for identification. This may be easily done by pasting on to each animal's hip, by means of ordinary flour paste, a small piece of common sheeting cloth upon which have previously been stamped consecutive numbers. Of course, this will not be necessary when the herd is small or known to the operator. Water and feed along toward evening, from 4 to 6 P.M.

Having determined, and stabled and marked, all animals that are to be tested, then begin taking the temperature. This is to be done with the ordinary thermometer, in the usual way, per rectum. It might be well to state here that one should have at least two or more thermometers that will register the same, so that in case of the accidental breaking of one or more the testing may not be interrupted. It is a very good practice to attach a string to the thermometer that is long enough, so that when the thermometer is inserted in the rectum the string can be wrapped around the tail a few times, so that, should the animal have a passage, which is very often the case, the string will hold the thermometer and prevent its falling to the floor and being broken. In case the thermometer is thrown out, it should never be reinserted until it has been properly adjusted, for very often the jar in falling will throw the mercury into the stem so that it will register 105° to 110°, and consequently there would be a faulty reading. This is quite likely to be the case where the string is attached between the bulb and stem of the thermometer.

It would be much handier for this kind of work if the thermometer were made with a bulb or metal ring at the end of the stem, so that a string could be attached there instead of being attached near the mercury bulb, thus necessitating the introduction of the string and knots into the rectum.

Another thing the operator should be prepared with is an old suit of clothes and rubbers, for the work is anything but clean.

As the temperature is taken, be careful to correctly read the register each time, and be as particular to correctly record the reading in a book previously prepared.

The form which I have adopted is somewhat as follows:

No.................... Breed.............................. Sex of animal..............................

Age .................. Weight ............................ Calved when ..............................

| Before injection. | | | | After injection. | |
|---|---|---|---|---|---|
| Time. | Temp. | | | Time. | Temp. |
| | | Am't injected........... Time of injection | .............. | | |
| | | Maximum temp. before injection, | ................ | | |
| | | " " after " | ................ | | |
| | | Higher before or after " | ................ | | |
| | | Average before " | ................ | | |
| | | " after " | ................ | | |
| Average | | Difference between averages | ................ | Average | |

The object of taking the temperature before making the injection is to get the normal, or more properly speaking, to get the maximum temperature of the animal.

In order to do this the temperature should be taken at least three times, morning, noon, and evening, but it is better to take it every two or three hours during the day, beginning at 8 or 9 A.M., and make the injection some time during the evening, depending somewhat upon the time one desires to begin work on the following morning, for it is not necessary to take the temperature during the first eight or ten hours after the injection has been made, for in case of a reaction the temperature rarely reaches the maximum height before the tenth hour after the injection was made. Usually from 8 to 10 P.M. is the best time, then one can resume taking temperature at from 6 to 8 A.M. the following morning.

Many, notably the Massachusetts Cattle Commission, according to report for 1894, and the New York State authorities, take the temperature but once or twice the same evening of making the injection.

But I am of the opinion that this is not a scientific or safe method, because of the amount of variation in the normal temperature of cattle during twenty-four hours. We are told that cattle usually register a higher temperature in the evening, but this has not been my experience, for the record of sixty-two head that I have assisted in testing forty-two head registered the maximum temperature between 8 A.M. and 5 P.M., while but twenty head registered the maximum between 6 and 12 P.M. In the majority of these cases the difference was insufficient to be misleading, but in ten head it amounted to from 1° to 2.2°. For illustration:

| | Before injection. | | | | | | After injection. | | | | | | Evening. | Maximum. | Difference. |
|---|---|---|---|---|---|---|---|---|---|---|---|---|---|---|---|
| | A. M. 9 | A. M. 10. 30 | P. M. 12. 30 | P. M. 2. 30 | P. M. 4. 30 | P. M. 8. 30 | A. M. 7. 30 | A. M 9. 30 | A. M. 11. 30 | P. M. 1. 30 | P. M 3. 30 | P. M. 5. 30 | | | |
| No. 12 | 102.2° | 101.4° | 103.4° | 103.8° | 102.0° | 102.3° | 102.2° | 102.4° | 102.8° | 103.0° | 102.8° | 101.6° | 102.3° | 103.8° | 1.5° |
| " 23 | 102.2 | 102.6 | 102.6 | 104.2 | ...... | 102.0 | 101.0 | 102.0 | 102.8 | 103.0 | 103.4 | 103.0 | 102.0 | 104.2 | 2.2 |

No. 12. Maximum temperature at 2.30 P.M. 103.8°, or 1.5° higher than at 8.30 P.M. 102.3°.

No. 23. " " at 2.30 P.M. 104.2°, or 2.2° " " at 8.30 P.M. 102.0°.

You will notice that in neither of these cases did the temperature rise as high during the second day as it did during the first day.

But suppose we had only taken the evening reading, which in No. 12 was 102.3°, and at 2.30 P.M. the second day it had registered 104.3°, or 5° higher than what it did register at that hour on the first day, which is quite likely to be the case, even in healthy animals, or in No. 23, whose evening register was 102°, had risen to 104.2° at 1.30 P.M. the second, or the same as it was at 2.30 P.M. the first day, we would have had, according to the readings, a difference of 2° in No. 12 and 2.2° in No. 23, which would be considered a reaction and the animals condemned, yet the animals may have been entirely free from the disease as were these animals, and as a result it would have been held that the test had failed in these two cases; but it would not have been the fault of the theory, or for that matter the practicability of the test, but it would have been the fault of the operator, who had not properly performed his work.

I have only cited these cases, yet I have never tested a herd when there was not one or more similar cases.

In the annual report of the Board of Cattle Commissioners of Massachusetts for 1894 is recorded the following cases:

Page 207: 10 P.M., 102.4°; following day, 9 A.M., 102.2°; 11 A.M., 103°; 2 P.M., 103.2°; 3 P.M., 104.3°; 5 P.M., 104.2°. Page 262: 6 P.M., 101.2°; following day, 5 A.M., 103°; 7 A.M., 103°;

9 A.M., 102.2°; 11 A.M., 102°; 1 P.M., 102°; 3 P.M., 102°. Page 263: 6 P.M., 102°; following day, 5 A.M., 102.1°; 7 A.M., 103.3°; 9 A.M., 104°; 11 A.M., 103.2°; 1 P.M., 102.1°; 3 P.M., 102.2°. These animals were condemned, but neither showed tubercular lesions on necropsy. On pages 267, 280, 287, 303, and 307 are recorded similar cases.

Now, in the light of my experience, I am led to believe that had the temperature of these cases been taken four or five times during the first day these errors would not have been made. In this same report, on pages 265, 5 cases; 266, 1 case; 267, 1 case; 268, 1 case; 280, 1 case, and 283, 2 cases are recorded where the maximum temperatures, after injection, registered from 105° to 107°, and giving an apparent reaction varying from 3.1° to 6.9°, which showed no lesions of the disease on necropsy. As these animals were tested at Brighton, where they had but recently arrived, we are led to believe that the high temperatures were due, in part at least, to some diseased condition incident to transportation, and not due wholly to the action of the tuberculin.

This leads to another thought, and that is, do not attempt to test animals that are in a fevered condition, for a rise of the temperature, due to the fever, might easily be mistaken for, and considered as, a rise due to the tuberculin.

Having taken the temperature a sufficient number of times the next step is to make the injection. This is done with an ordinary graduated hypodermic syringe, which has been properly sterilized, then set the gauge on the piston so that no more than the amount wanted can be injected. The exact size of the dose used varies with various operators, some using 3 c c. of tuberculin solution for an average-sized animal, and more for bulls and large animals. I have always used 1 c.c. of tuberculin solution for every 500 pounds animal weight (approximately). Of course, one must usually use his judgment as to the weight, as it is not practical, if it were possible, to weigh each animal.

Dr. Reynolds, of the Minnesota Experiment Station, has had just as good results when he used 1 c.c. tuberculin solution for every 750 pounds animal weight, but as one is liable to err more or less in estimating the weight, I am inclined to favor the 1 c.c. for every 500 pounds animal weight.

One can during the day form his estimates of the weights of each animal, and from that form his dosage and mark the same in his record book.

It might be well to state here that each syringe should be tested, so that its graduation will be correct.

If you have any number to inject you should have two trustworthy assistants, one to make the injections, the other to read off from the record book the previously prepared dosage for each animal, while you fill the syringe and set the gauge.

In this case you should have at least two syringes and a good supply of extra needles, so as not to be delayed in case of accidentally breaking one or more.

It does not matter much into which part of the body the injection is made, but usually the shoulder is the most convenient, and, considering the ease with which cattle use their hind feet, it is the safest.

The one making the injection walks up to the side of the animal, and, if it be not too large, leans over the animal and pinches up the skin of the shoulder on the opposite side with one hand, and introduces the syringe needle with the other, being careful not to push the needle into the muscular tissue underneath, for then the animal will not stand quiet, which it will usually do if the needle be properly introduced.

A point that always demands careful attention is the needle, that it does not become clogged, and thus forcing the tuberculin out at the coupling or up the barrel of the syringe past the piston. This mishap can be easily remedied if it is noticed at first, but could not if the same needle had been used in two or three animals before it was noticed, for it would not be known whether one or more had missed the injection, consequently the syringe and needle should be examined after each injection.

Having finished making the injections nothing further need be done for eight to ten hours; then the cattle should be put in as near the same condition as they were on the first day, and the temperature readings be continued at intervals of two to three hours for eight to ten hours. Thus, if you begin the second day at 6 to 8 A.M. the readings and their records should be continued until 5 or 6 P.M.

In case there should be any marked difference, either a rise or fall in the temperature at any time during the testing, it should be taken a second or third time so as to correct any error. As the work progresses the second day you will be able to derive correct conclusions, as all the animals showing a rise of 2° or more are said to have reacted and are diseased, and should be condemned.

There may be said to be more or less question as to just what constitutes a reaction. What we are to understand as a typical reaction is where there is a gradual rise in the temperature on the second day, from normal in the morning to a height of 2° or more above the maximum of the first day, and then a gradual falling to the normal; for illustration in Bulletin No. 7 of the Bureau of Animal Industry, Washington, D. C., page 63, animal No. 316, we find the following record:

| | | | | | | | | | | | | | | |
|---|---|---|---|---|---|---|---|---|---|---|---|---|---|---|
| Time . . . . | A. M 9 | A. M. 10 | A. M. 11 | M. 12 | P. M. 1 | P. M. 2 | P. M. 3 | P. M. 4 | P. M. 5 | P. M. 6 | P. M. 7 | P. M. 8 | P. M 9 | P. M. 10 |
| Before 1st day | 101.3° | 101.4° | 101.6° | 101.8° | 101.6° | 101.6° | 102.2° | 102.6° | 101.4° | 101.5° | 101.4° | 101.6° | 101.4° | 101.2° |
| After inj. 2d day | 101.4 | 101.4 | 100.6 | 101.0 | 101.3 | 101.4 | 101.6 | 102.4 | 102.0 | 102.6 | 103.2 | 103.7 | 105.3 | 105.9 |
| Time . . . . | P. M. 11 | M. 12 | A. M. 1 | A. M. 2 | A. M. 3 | A. M. 4 | A. M. 5 | A. M. 6 | A. M. 8 | A. M. 9 | A. M. 10 | M. 12 | P. M. 1 | |
| Before 2d day | 101.5° | 100.8° | 100.8° | 101.3° | 101° | 100.8° | 101.8° | 101.5° | 101.4° | Inj. | ...... | ...... | | |
| fter inj. 3d day | 106.2 | 106.c | 105.4 | 105.2 | 105 | 104.8 | 103.2 | 102.8 | 102.7 | ...... | 101.8° | 102.5° | 102.3° | |

This may be considered a typical reaction and is cited because the temperature was taken every hour for the extended period of forty-seven hours, then three times more during the next five ensuing hours, covering in all a period of fifty-two hours. Here we have a gradual rise from 102° at 5 P.M., or, eight hours after the injection, was made, to 106.2° at 11 P.M., or, twelve hours after the injection was made, then a gradual fall in the temperature until the normal is reached at 10 A.M. the following day, or twenty-five hours after the injection was made.

In this case the maximum temperature of the reaction was at the fourteenth hour after the injection, while in a record of eighty-five cases taken from the Bulletin of the Iowa Experiment Station the time of maximum registers varied from the tenth to the twentieth hour, with an average of $15\frac{16}{17}$ hours after the injection, from which we learn that the readings should be continued from the tenth to the twentieth hour from the time of making the injection. But in practice it would not be necessary to follow up the readings later than the sixteenth or eighteenth hour unless there was some appearance of a reaction, by the beginning of a rise which would begin to make its appearance at the sixteenth, or, more especially, the eighteenth hour; nor is it necessary to continue the readings upon animals that have plainly shown a reaction, except you wish to study the reaction, which is advisable when convenient, for in practice a typical reaction may be said to be the exception rather than the rule.

But in cases where the maximum temperatures of second day is not more than 1° or 2° above the maximum of the first day, it may become necessary to follow up the readings for a longer period of time, and should it then give a typical reaction, although the difference of the maximum may not have been more than 1°, as the following case will show, taken from the records of a test made by Prof. W. B. Niles and myself.

| Time . . . . . . . . | A. M. 9 | A. M. 10 | A. M. 12. 30 | P. M. 2. 30 | P.M. 4. 30 | P.M. 8. 30 | Average. | Maximum. |
|---|---|---|---|---|---|---|---|---|
| Animal No. 8, before injection . . | 101. 8° | 100. 4° | 103. 8° | 108. 8° | 102. 2° | 114. 4° | 102. 7° | 104. 4° |
| Injection made 8.30 P.M. Time . . | A. M. 7. 30 | A. M. 9. 30 | A. M. 11. 30 | P.M. 1. 30 | P.M. 3. 30 | P.M. 5. 30 | | |
| Second day, or after injection . . | 100. 4° | 103. 4° | 103. 4° | 105. 4° | 105. 4° | 105. 0° | 103. 7° | 105. 4° |

In this case the difference between the maximum temperature of the first and second days, also the average of the two days, was 1°, but from the character of the reaction she was condemned, and a necropsy verified the correctness of the diagnosis.

A further analysis of this case showed that the animal registered a very high temperature at 8.30 P.M. the first day, the cause of which we were unable to discover. Some might think that it was the result of an error in the reading, but this is not the case, as the temperature was taken the second time with a different thermometer. And I might here say that I always take a second and sometimes a third reading whenever the readings are out of the ordinary and unusually high, and this should be done, thereby avoiding any errors either in the reading or owing to a false registration of the thermometer. Professor Osgood has noted that in some cases where the disease has reached a very advanced stage, that instead of there being a rise in the temperature there will be a fall, it will gradually drop considerably below normal, and then gradually regain it, in which case the reaction would consist in a lowering of the temperature instead of a rise.

It is not within the province of this paper to discuss the action of tuberculin further than it is concerned in the test, but questions are frequently asked, such as "Will the tuberculin have any bad effects upon healthy animals?" The answer is "No."

In a set of experiments recorded in Bulletin No. 82 of the New York Experiment Station, Professor James Law clearly demonstrated that tuberculin not only has no deleterious results on the healthy animal, but that it has no marked affect on the milk flow. It will not cause any disease when properly prepared. As to its action upon tuberculous animals, we have but little concern further than in its use as a diagnostic agent, yet experiments tend to show that it has very little effect either to aggravate latent cases of the disease or to produce resolution.

Professor E. Klebs claims that by treating the ordinary tuberculin of Koch with an acid solution of soda iodide of bismuth he obtains a solution which he calls antiphthisin that is free from the toxic properties of tuberculin, and that it will destroy the tubercle bacillus, but as yet this theory is in its infancy, and the most we can do is to wait and watch for results.

To recapitulate: The operator should be supplied with a sufficient quantity of a reliable preparation of tuberculin, two or three extra thermometers, one or more properly graduated hypodermic syringes, with extra needles, so that any accidental breakage will not delay the testing. He should also have the proper clothing.

See that all animals are in a proper condition for testing. Then be scrupulously careful in your readings, which should be frequent enough to avoid any errors due to the erratic temperature, and just as careful in recording the results of your work. In short, consider the procedure in its true light, which is a purely scientific one, and conduct it on scientific grounds, and the results will not deceive you.

In making necropsies never give up in despair of finding tubercular lesions until the entire carcass has been completely dissected, for no part of the animal economy is exempt from the ravages of the tubercular bacillus. The lungs and lymphatic system are the most frequently affected, yet if these organs are found free from the disease it does not signify that the animal had been free from the disease, for any of the other organs and tissues may be the seat of the affection.

The only thing lacking in tuberculin to make it an ideal diagnostic agent in the lower animals is that the reaction gives no indication of the extent or localization of the pathological lesions.

